# Critical analysis of methods to determine growth, control and analysis of biofilms for potential non-submerged antibiofilm surfaces and coatings

**DOI:** 10.1016/j.bioflm.2024.100187

**Published:** 2024-02-27

**Authors:** J. Redfern, A.J. Cunliffe, D.M. Goeres, N.F. Azevedo, J. Verran

**Affiliations:** a Department of Natural Sciences, Faculty of Science and Engineering, Manchester Metropolitan University, UK; b Center for Biofilm Engineering, Montana State University, MT, USA; c LEPABE – Laboratory for Process Engineering, Environment, Biotechnology and Energy, Faculty of Engineering, University of Porto, Rua Dr. Roberto Frias, 4200-465, Porto, Portugal; d ALiCE – Associate Laboratory in Chemical Engineering, Faculty of Engineering, University of Porto, Rua Dr. Roberto Frias, 4200-465, Porto, Portugal; e Department of Life Sciences, Faculty of Science and Engineering, Manchester Metropolitan University, UK

## Abstract

The potential uses for antibiofilm surfaces reach across different sectors with significant resultant economic, societal and health impact. For those interested in using antibiofilm surfaces in the built environment, it is important that efficacy testing methods are relevant, reproducible and standardised where possible, to ensure data outputs are applicable to end-use, and comparable across the literature. Using pre-defined keywords, a review of literature reporting on antimicrobial surfaces (78 articles), within which a potential application was described as non-submerged/non-medical surface or coating with antibiofilm action, was undertaken. The most used methods utilized the growth of biofilm in submerged and static systems. Quantification varied (from most to least commonly used) across colony forming unit counts, non-microscopy fluorescence or spectroscopy, microscopy analysis, direct agar-contact, sequencing, and ELISA. Selection of growth media, microbial species, and incubation temperature also varied. In many cases, definitions of biofilm and attempts to quantify antibiofilm activity were absent or vague. Assessing a surface after biofilm recovery or assessing potential regrowth of a biofilm after initial analysis was almost entirely absent. It is clear the field would benefit from widely agreed and adopted approaches or guidance on how to select and incorporate end-use specific conditions, alongside minimum reporting guidelines may benefit the literature.

## 1 Introduction

The presence and activity of microorganisms at surfaces or interfaces has significant economic impact [1] and is a widespread, multi-sector problem, with examples ranging from water-cooling towers [2], oil and gas production [3], paper and pulp processes [4] and healthcare (both the built environment [5] and medical devices [6]). These microorganisms can be deposited at low density but in appropriate conditions can grow rapidly to reach numbers capable of causing significant impact. These surface-attached microbial communities can collectively be considered biofilms, which can form at a surface-liquid interface (a submerged environment) or a surface-air interface (a non-submerged environment). More recently, non-surface adhered aggregates have been acknowledged as biofilm, widening the classic definition [7]. Non-submerged environments which can support biofilm formation (also known as dry surface biofilms – DSB) are varied, and the presence of viable microorganisms is a significant problem for many healthcare, industry, and domestic environments [e.g. 8,9].

Management and control approaches to biofilm commonly fall into two categories: curative or preventative. Curative action is usually where an intervention results in the destruction, reduction, or removal of a biofilm, whereas preventative action typically prevents biofilm from forming in the first instance. Explicit curative action against biofilm is difficult, if not practically impossible, to achieve in locations such as the built environment and industrial processes, because multiple microbiological reinoculation events occurs over time. However, curative actions, such as mechanical, physical and chemical methods are widely used. For example, cleaning-in-place processes are established practices across industrial settings [10,11], e.g. physical cleaning with standard domestic products [12,13] and other physical methods such as plasma treatment and ultrasonication [14].

In addition to containing biocides, antimicrobial surfaces and coatings (e.g. nanoparticle metals) can exhibit other properties such anti-adhesion (e.g. topographical modifications) and contact-active action (e.g. enzymes, QACs), with such materials reviewed elsewhere in the literature [15] and further explored as part of this study. If these antimicrobial surfaces and coatings can prevent initial attachment of microorganisms, or kill those cells that do adhere, they would inherently be providing preventative antibiofilm activity. But if such an antibiofilm surface were to be deployed with an expectation of preventative action against biofilm, then test methods that are used to generate efficacy data should be scientifically rigorous, robust, and reproducible enough to demonstrate the intended antibiofilm activity in the intended application for a relevant length of time.

Efforts to encourage a standardised approach to biofilm research and antibiofilm efficacy exist in industrial communities, whereas academics have developed, adopted and reported best practices in the literature. Such minimum reporting information standards for experiments have included where sessile microbial communities exist on surfaces [16] and experiments that report spectrophotometric and fluorometric methods to assess biofilm formation [17]. In addition, recent papers have called for further work to standardise such methodology in biofilm research [18,19]. It is also widely acknowledged that vocabulary used to describe biofilms can be inconsistent. For industry, and particularly those where an antibiofilm claim may be regulated by a competent authority, standardised methods (and clearly defined terminology) developed by national and international standards bodies such as the International Organization for Standards (ISO), European Committee for Standardization (CEN), British Standards Institute (BSI) and ASTM International among others, are essential.

There are numerous challenges when creating and validating standardised methods. For example, significant experimental work is required to assess different approaches and methodologies, and gather enough data to ensure methods are reproducible, reliable, and repeatable. Such methods need to be appropriate for their intended users (e.g. industry laboratories) whilst still generating appropriate data applicable support their intended claim. These methods need appropriate statistical certainty and often take years before being adopted by a standards setting organisation. Ideally, these methods should be designed with regulatory bodies in mind and ensure that they fulfil any requirements they may have, which are often considered on a case-by-case basis and can vary from region-to-region. Never-the-less, progress to develop standards around biofilm to make ‘antibiofilm’ claims is on-going. For example, standardised methods are available that relate to antibiofilm activity, predominantly assessing the performance of a liquid disinfectant to kill (curative action) a matured biofilm (e.g. ASTM E2799-22, ASTM E2871-21). However these methods are not intended to demonstrate prevention or retardation of a biofilm by an antimicrobial surface or a preventative treatment – particularly in non-submerged environments. ASTM Method E3321-21 is an example of a standard that does assess efficacy of an antimicrobial surface, and was approved to determine the efficacy of antimicrobial urinary catheters against biofilm (which by its nature can be a submerged environment). However, E3321-21 is specific and was not designed to be used to test antimicrobial surfaces found in the built environment. For novel antibiofilm surfaces and coatings to contribute to the challenges of biofilm control in medical, domestic and industry settings, developing a standardised method that can be trusted to generate translatable data is critical. Recently, ISO 4768:2023 ‘measurement method of anti-biofilm activity on plastic and other non-porous surface’ describes a method intended as a screening tool for material development, using crystal violet to stain cells (excluding textiles or photocatalytic materials). More work is required to understand the biological and applied/in-situ requirements of such biofilm-associated standards, including a recognition of the diverse, regulated industries which require bespoke standards, and the challenges of harmonisation in different international markets. Whilst creating biofilm-associated standards is not a simple or quick task, the development of methods that enable users to be confident of their antibiofilm data is essential, and beneficial to those stakeholders at all stages of developing and using such antibiofilm products.

This review aims to understand the methodological approaches to test the efficacy of antimicrobial surfaces used to prevent biofilm, and to consider them within the context of definition/terminology, experimental design, challenges posed by biofilm testing (e.g. methods to quantify biofilm), and parameters and required evidence for ‘antibiofilm’ claims. It is hoped that findings will help inform the development of more robust yet flexible standardised methods, provide guidance and suggest experimental testing approaches in the context of point-of-use application.

## 2 Method

### 2.1 Search and sorting articles

A review was conducted to understand the methodological approaches described in the literature that were used to determine the efficacy of surfaces or coatings that may be claimed as antibiofilm. Using the Scopus database, the following terms were searched across journal titles, abstracts and keywords: "antibiofilm” OR "*anti*-biofilm” AND coating OR surface OR material OR paint OR interface. There were no restrictions on date of publication. The search returned 3934 articles, reduced to 3411 once filtered to remove non-research articles (e.g. reviews, book chapters etc). These remaining papers were exported as CSV format, containing citation information, bibliographic information, abstract & keywords, funding details and other information. All titles and abstracts were read to filter for scope. An article that described a material that was (i) not a surface, (ii) exclusively describing a medical device or (iii) exclusively describing a material for a submerged application (e.g. ship hull), was excluded from further analysis. If an article described a non-built environment application (e.g. medical) but also explored uses in other applications (e.g. built environment) they were included in further analysis. Following this process, the final selection of papers for analysis was 78. Most papers excluded from the analysis focused on antimicrobial compounds, nanomaterials or other innovations that were not a coating and were suspended in solution with potential activity against a biofilm that had formed on a surface (such as a 96-well plate). From here-on-in, due to the variety of terminology used within the articles, this review will use the term ‘surfaces’ to describe the materials. Previously described categories (*vide supra*) of antimicrobial surfaces were used to group the surfaces in the reviewed research articles [15].

### 2.2 Bibliometric analysis

Bibliometric analysis was carried out using R (v4.2.3) via RStudio (v3.4.1) with package Bibliometrix (v4.0.0) and associated Biblioshiny app [20]. The CSV file generated from the Scopus search was edited to only include the 78 papers included in the final analysis and used as the input file. Data generated from Bibliometrix/Biblioshiny were downloaded for analysis. To understand the academic discipline with which the authors may be aligned, the name of the department with which each author was affiliated with was manually recorded and categorised into whichever of the following discipline categories that most likely reflected their department name: microbiology, biology (including biotechnology and bioengineering), chemistry, engineering, environmental sciences, food, materials science, medical/health, physics, interdisciplinary or undefined/non-specific.

### 2.3 Reviewing the methods described in the articles

Prior to starting the search, the authors decided on a series of questions through which to interrogate, extract and analyse the information described in the articles. This process was designed to provide insight into methodological approaches used to assess antibiofilm activity of surfaces. These were.

• is a definition of biofilm present?
• what type of material is being tested?
• what type of antimicrobial action is exhibited, if any?
• what method was used to form the biofilm (including environment)?
• what species of microorganism were used?
• how was biofilm quantified?
• were adequate positive/negative controls used?
• how were bacteria remaining on the surface quantified (if at all)?
• was regrowth of biofilm assessed?
• does the article claim success for an antibiofilm surface?
• what are the requirements for passing as an anti-biofilm material?
• did the experiment contain repeats or replicates and how were they described?

These criteria were used to create a review matrix to aid with data collection, analysis and interpretation.

## 3 Results

### 3.1 Bibliometric analysis

**Fig. 1:**
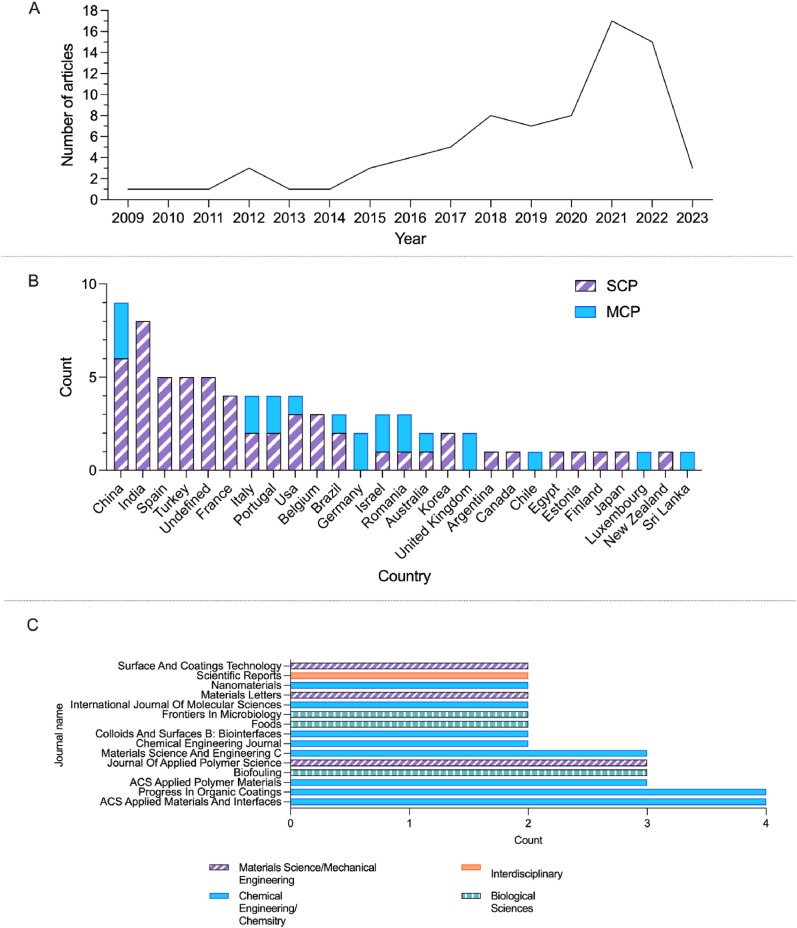
Overview of bibliometric data. A) Number of research articles included in the analysis split by year of publication (data obtained April 2023, as such 2023 publication numbers are lower than 2022 despite upward trend). B). The number of research articles that either had authors from a single country (SCP) or authors from multiple countries (MCP), split by the corresponding authors designated country. C). The number of research articles included in the analysis that featured in the 15 most frequently used journals. Colours/patterns represent the subject area to which the journal aligns.

Publication date of articles ranged from 2009 to 2023, across 55 journals. The number of research articles increased over time, with over half of these published since 2020 (Fig. 1A). The majority of the research articles were single country publications (i.e. authors were all from the same country), with authors from China and India producing the highest number of publications included in the analysis (Fig. 1B). Annual growth rate of the number of research articles in this analysis was 8.16%, with an average citation per article of 17.62. Of the journals that featured two or more of the research articles (n = 15, Fig. 1C), the majority were focused on the disciplines of engineering and chemistry, with three focused on biological sciences, of which only one was overtly microbiology. Of all the journals that featured articles within this review (n = 55), seven were overtly microbiology in focus. Analysis of author department affiliation (n = 571) showed the assumed (based on departmental affiliation) author-aligned discipline was chemistry (30.1%), followed by materials science (9.3%), biological sciences excluding explicit microbiology (8.8%), engineering (7.7%), physics (6.8%), food (6.5%), medical/health (6.3%), microbiology (6.1%) and environmental sciences (4%), whilst 8.9% where interdisciplinary and 5.3% were undefined/non-specific.

### 3.2 Type of surface and antimicrobial action

The surfaces, and their modes of actions that were assessed for antibiofilm activity were diverse. Most materials were categorised as biocide releasing (n = 36) or contact active (n = 32) with fewer antiadhesive (n = 10). Biocide releasing surfaces were predominantly reported as using inorganic nanoparticles and powders such as Ag [[21], [22], [23], [24], [25], [26], [27], [28], [29], [30], [31], [32], [33]], Cu [[34], [35], [36], [37], [38]], Ti/TiO_2_ [[39], [40], [41], [42], [43], [44], [45]], ZnO [[46], [47], [48]], Cu-Zr-Ni [49], CeO_2_ [50], Ag/TiO_2_ [44,[51], [52], [53]], Fe/Fe_3_O_4_ [44,51], TaO_x_N_y_ [54] as well as loaded-metal organic frameworks (MOFs) [55], and antibiotics such as gentamicin [56]. Contact active surfaces utilized a range of approaches including plasma [[57], [58], [59], [60], [61], [62], [63], [64], [65], [66], [67], [68], [69], [70], [71], [72]], amino-acids, polypeptides, enzymes [[73], [74], [75], [76], [77], [78], [79], [80], [81], [82], [83], [84]], hormones [85], neurotransmitters [86] and polymers or copolymers [[57], [58], [59], [60], [61], [62], [63], [64], [65], [66], [67]]. Antiadhesive approaches included nanospikes [87], electrochemical etching [88], patterning [89], hydrophobic modifications [[90], [91], [92], [93]], and included polymers [[94], [95], [96]], metals [97] and coatings using novel approaches such as candle soot [98].

### 3.3 Definition of biofilm

When analysing the text of the articles, 34% (n = 27) did not provide a definition of biofilm within the introduction or method sections. Where definitions did exist, they tended to focus on the problem/impact that biofilm can have (such as medical and industrial) and the classic model of biofilm formation. In particular, the most common words related to bacteria, surfaces, extracellular polymeric substance and attachment and colonisation. Whilst research articles are generally consistent in the use of the term antibiofilm (with some disparity between antibiofilm and anti-biofilm – which could affect bibliometry), a small number use the term biofouling and biofilm interchangeably.

### 3.4 Methods for forming the biofilm

**Fig. 2:**
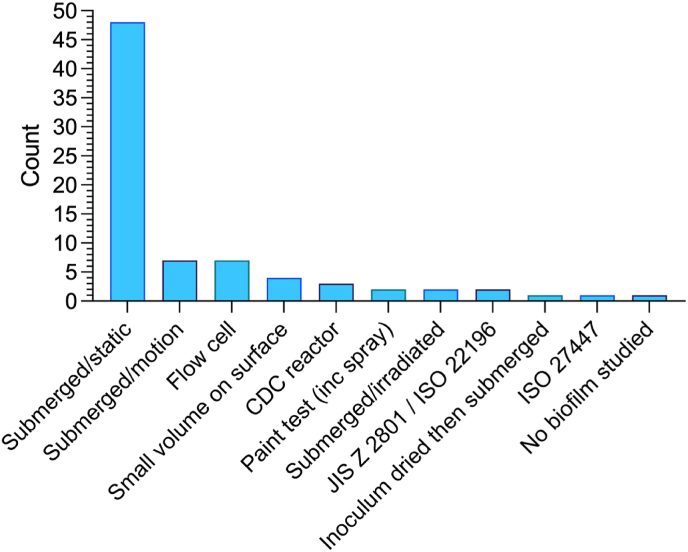
-Biofilm formation methods described in research articles (n = 78).

Whilst methods for forming biofilm on surfaces were categorised into eleven different approaches (Fig. 2), submerging a material in growth media inoculated with a microorganism and incubating with no motion (static) was the predominant approach (n = 48, 62%) for example in multi-well plates. Some research articles used a similar submerged set-up but had their incubation with orbital motion (n = 7, 9%) whilst others described submerged systems where a flow of incubating media passed over a surface, such as flow cell systems (n = 7, 9%) and the CDC reactor (n = 3, 4%). Application of microorganisms in liquid applied directly to the surface and incubating (n = 3, 3%) either with or without additional media, or first allowing a droplet (10 μL) to dry at room temperature in 30 min followed by submersion and incubation (n = 1, 1%), or allowing a droplet (30 μL) to dry followed by quantification were also described (n = 1, 1%). Standardised methods for assessment of antimicrobial activity of a surface against planktonic culture JIS Z 2801/ISO 22196 (n = 2, 3%) and ISO 27447 (n = 1, 1%) were also used. Despite suggesting a surface has antibiofilm activity, one article did not use any microorganisms in their article (n = 1, 1%).

### 3.5 Experimental considerations

Within the different methodologies described above, experimental variables and other considerations were varied and wide-ranging. With regards to technical replicates (a test performed on the same sample type multiple times within one experiment) and biological repeats (where experiments are identical in methodology but with biologically distinct inoculum) to assess variation within experiments, 29 articles (37%) did not describe either, but 14 (18%) of these provided figures or tables that demonstrated variation (e.g. error bars) that indicated replicate testing was undertaken. Where the method did include such information, the most common approach was to use three technical replicates (28%, n = 22), with others describing using 4 (4%, n = 3), 2 (1%, n = 1) or 6 (1%, n = 1) technical replicates. Two articles described only using either 2 (1%, n = 1) or 3 (1%, n = 1) biological repeats with no technical replicates. Seven articles described both technical replicates and biological repeats, describing 3 replicates and 3 repeats (6%, n = 5), 4 replicates and 4 repeats (1%, n = 1) or 2 replicates and 2 repeats (1%, n = 1). Thirteen articles described their approach to experimental variability but used imprecise or vague language. For example, stating ‘up to’ or ‘more than’ several replicates, or using statements that make it difficult to assign their approach as either technical replicates or biological repeats.

There were 31 different media described to support biofilm formation/growth as part of antibiofilm assessment across the research articles a total of 81 times (Table 1). Those media included commercially available microbiological media (39%, n = 11), and variation of these (35%, n = 8) (e.g. reduced strength or supplementation with a sugar) were most common, including a range of media considered nutrient-rich through to minimal media. Other media were described as a mixture of chemicals (13%, n = 4) e.g. yeast extract in PBS. Additionally, there were three instances (10%) of application specific media (synthetic tap water and tap water), and one instance (3%) of mammalian cell culture media.

**Table 1:** –Media described as part of the biofilm growth/formation method within the 78 research articles.

Standard microbiology media	*Count*	Modified microbiology media	*Count*	Chemical recipe	*Count*
Luria-Bertani (LB) broth	18	BM2 minimal media +0.4% glucose	2	PBS with yeast extract	1
Nutrient Broth (NB)	12	1% TSB	2	PBS	1
Tryptone Soya Broth (TSB)	11	1% TSB + glucose	2	3% NaCL peptone water	1
Brain Heart Infusion (BHI) broth	5	M63 media with glucose and casamino acids	2	Yeast extract, sodium nitrate + glucose	1
Mueller Hinton broth (MHB)	5	20% TSB	1		
M9 minimal media	2	50% MHB	1	**Application specific media**	***Count***
CT media	1	10% TSB + 0.3% TSB	1	Tap water	1
Sabouraud dextrose broth (SDB)	1	LB + 10% LB	1	Synthetic tap water	1
Tris minimal media	1	LB broth containing 1 wt% glucose and 1 wt% NaCl	1	Potable water	1
Yeast extract, peptone and dextrose (YPD) media	1	NB with 1 wt% dextrose	1		
Yeast nitrogen base + glucose (YNBG)	1	TSB + Glucose	1	**Mammalian cell media**	***Count***
Yeast peptone dextrose (YPD)	1			Roswell Park Memorial Institute (RPMI) 1640 media	1

**Fig. 3:**
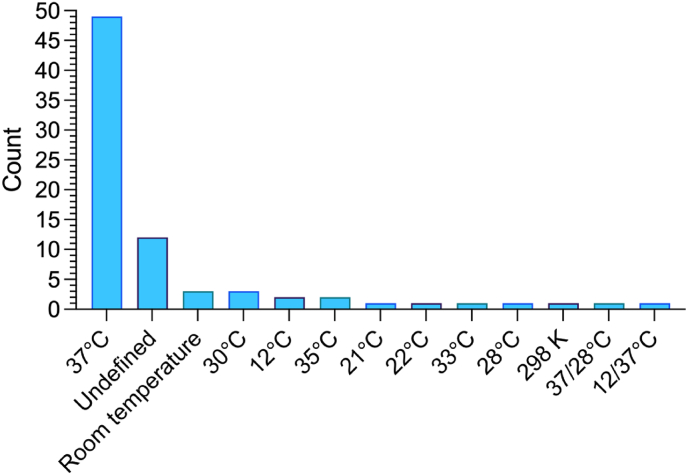
Incubation temperatures described for the biofilm formation stage of assessing antibiofilm surfaces. Temperatures 37/28 °C were described due to the use of different microorganisms and 12/37 °C were described due to the use of using different temperatures for different amounts of time across the experiment. Any methodology that omitted the temperature or for which it was not clear was categorised as undefined. N = 78.

Thirteen different temperature conditions were reported across the studies (Fig. 3), with the majority (63%, n = 49) of research articles reporting biofilm formation/incubation at 37 °C. Fewer research articles did not define temperatures - including 12 studies not reporting a temperature at all (15%, n = 12), and three studies reporting ‘room temperature’ (4%, n = 3). The remaining articles provided various incubation temperatures including 30 °C (4%, n = 3), 35 °C (3%, n = 2), 12 °C (3%, n = 2), 21 °C (1%, n = 1), 22 °C (1%, n = 1), 24.85 °C (reported at 298 K, 1%, n = 1), 28 °C (1%, n = 1), and 33 °C (1%, n = 1). Two articles reported two different temperatures, 37/28 °C were used due to different microorganisms that required different temperatures (1%, n = 1) and 12/37 °C were used due to experimental design requiring a long incubation time (1%, n = 1). There were only three articles that explicitly described temperature selection to be associated with intended end-use of the antibiofilm material, which described the temperature of 12 °C as maximum ambient temperature for processing food of animal origin as dictated by European Union Regulation 853/2004/EC (these are the only direct references to regulatory considerations in biofilm methodology in the entire collection of 78 papers).

**Fig. 4:**
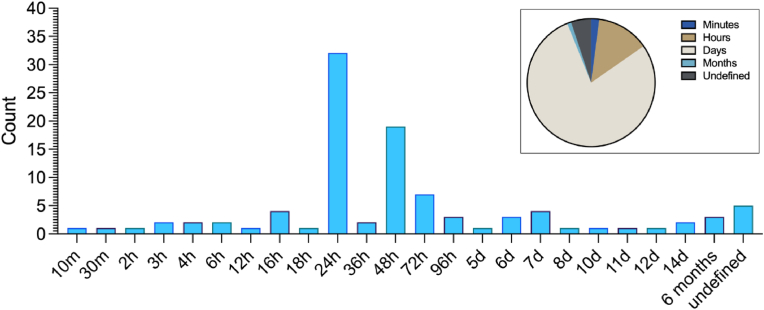
Incubation time described for the biofilm formation stage of assessing antibiofilm surfaces. Where more than one time is reported in a research article, they are reported individually. M = minutes, h = hours, d = days. Any. Insert pie chart (right) represents incubation times categorised into minutes, hours, days (starting at 24 h), months or where methodology that omitted the incubation time or for which it was not explicit was categorised as undefined. n = 98.

Across all research articles, 23 different biofilm incubation times were reported, ranging from 10 min to 6 months, and five articles did not explicitly report an incubation time (Fig. 4). The most frequently described incubation times were 24 h (32%, n = 32), 48 h (19%, n = 19), 72 h (7%, n = 7) and 16 h (4%, n = 4). Other time points reported for biofilm incubation included six months (3%, n = 3), 10 min (1%, n = 1), 30 min (1%, n = 1), 2 h (1%, n = 1), 3 h (2%, n = 2), 4 h (2%, n = 2), 6 h (2%, n = 2), 12 h (1%, n = 1), 16 h (4%, n = 4) and 18 h (1%, n = 1). A small number of articles reported incubation times lasting several days.

There were 82 microbial cultures used to assess antibiofilm properties of surfaces. These microorganisms were diverse, comprising numerous isolates (e.g. hospital isolate) or strains (e.g. had a culture collection ID) relating to 27 different species. These consisted of Gram-negative bacteria (51%, n = 42), Gram-positive bacteria (35%, n = 29), yeast (7%, n = 6) and moulds (6%, n = 5) (Table 2). In total, there were 147 instances of microorganisms named as some articles studied more than one species. Of the Gram-negative bacteria, there were 13 species, including *Escherichia coli*, *Pseudomonas aeruginosa*, *Klebsiella pneumoniae*, *Proteus* sp, *Shewanella putrefaciens*, *Salmonella* sp, *Acinetobacter baumannii*, *Pectobacterium carotovorum*, *Legionella pneumophila* and *Serratia marcescens* described a total of 73 times. Of Gram-positive bacteria there were 8 species, including *Staphylococcus aureus* (and MRSA), *Listeria monocytogenes*, *Bacillus* sp., and *Streptococcus mutans* mentioned a total of 61 times. There were only two species of yeast described, including 5 isolates of *Candida albicans* and 1 isolate of *Candida tropicalis*. Four species of mould were described, including 2 isolates of *Apergillus niger*, and 1 isolate each of *Aspergillus ochraceus*, *Cladosporium cladosporioides* and *Penicillium expansum*. Whilst type strain/isolate identifying information (e.g. culture collection reference number) are provided in most cases, with 26 instances where the microorganism is described with only their species name (e.g. *E. coli*).

**Table 2:** A list of microorganisms within each research article. Names are listed as described in the article including strain identifiers. Numbers in the count column represent the number of times the corresponding microorganism was mentioned across all articles (n = 146).

Gram negative bacteria	*Count*	Gram positive bacteria	*Count*	Yeasts	*Count*	Moulds	*Count*
*Escherichia coli*	6	*Staphylococcus aureus*	10	*Candida albicans* ATCC 10239	2	*Aspergillus niger* ATCC 16404	1
*Escherichia coli* DH5α	3	*Staphylococcus aureus* ATCC 25923	9	*Candida albicans* SC5314	2	*Aspergillus niger* CECT 2088	1
*Escherichia coli* 0157:H7	3	*Staphylococcus aureus* ATCC 6538	4	*Candida albicans*	1	*Cladosporium cladosporioides* CECT 2111	1
*Escherichia coli* ATCC 25922	3	*Staphylococcus aureus* ATCC 29213	2	*Candida albicans* ATCC 10231	1	*Aspergillus ochraceus* CECT 2093	1
*Escherichia coli* 25922	2	MRSA (medical isolate)	1	*Candida albicans* CAI4	1	*Penicillium expansum* CECT 2275	1
*Escherichia coli* ATCC 8739	2	*Staphylococcus aureus* Lux	1	*Candida tropicalis*	1		
*Escherichia coli* K12	2	MRSA CIP 103.811	1				
*Escherichia coli* CECT515	2	*Staphylococcus aureus* AB 136	1				
*Escherichia coli* MG 1655	2	*Staphylococcus aureus* ATCC 33591	1				
*Escherichia coli* ATCC 25404	1	*Staphylococcus aureus* CMCC (B) 26003	1				
*Escherichia coli* AB318	1	*Staphylococcus aureus* ATCC 12600	1				
*Escherichia coli* CMCC (B) 44102	1	*Staphylococcus aureus* ATCC 21351	1				
*Escherichia coli* IBEC 101	1	*Staphylococcus epidermidis* ATCC 35984	2				
*Escherichia coli* 10536	1	*Staphylococcus epidermidis*	1				
*Escherichia coli* 47076	1	*Staphylococcus epidermidis* ATCC 12228	1				
*Escherichia coli* ATCC 23501	1	*Staphylococcus epidermidis* NCTC 11047	1				
*Pseudomonas aeruginosa*	3	*Listeria monocytogenes* CECT 911	5				
*Pseudomonas aeruginosa* ATCC 10145	2	*Listeria monocytogenes* ULE1264	3				
*Pseudomonas aeruginosa* ATCC 15692	2	*Listeria monocytogenes* ULE1265	3				
*Pseudomonas aeruginosa* Lux	1	*Listeria monocytogenes*	2				
*Pseudomonas aeruginosa* MTCC 7815	1	*Listeria monocytogenes* CIP 10357	1				
*Pseudomonas aeruginosa* CIP 82.118	1	*Listeria monocytogenes* ATCC 7644	1				
*Pseudomonas aeruginosa* RRLP1	1	*Bacillus subtilis*	2				
*Pseudomonas aeruginosa* PA14	2	*Bacillus subtilis* 1904-E	1				
*Pseudomonas aeruginosa* ATCC 27853	3	*Bacillus licheniformis*	1				
*Pseudomonas aeruginosa* PAO1	5	*Bacillus cereus* (food isolate)	1				
*Pseudomonas aeruginosa* ATCC 25668	1	*Bacillus* sp. UFPEDA 189	1				
*Pseudomonas fluorescens* DMS 50090	1	*Bacillus subtilis* ATCC 6633	1				
*Klebsiella pneumoniae*	1	*Streptococcus mutans*	1				
*Proteus vulgaris*	1						
*Proteus mirabilis*	1						
*Shewanella putrefaciens*	1						
*Salmonella typhimurium* ATCC 14028	3						
*Salmonella typhi*	3						
*Salmonella typhimurium*	1						
*Salmonella*	1						
*Salmonella enteritidis* 706 RIVM	1						
*Acinetobacter baumannii* (medical isolate)	1						
*Acinetobacter baumannii* C80	1						
*Pectobacterium carotovorum subs. Carotovorum*	1						
*Legionella pneumophila* ATCC 33152	1						
*Serratia marcescens* ATCC 14756	1						

### 3.6 Assessing the biofilm

**Fig. 5:**
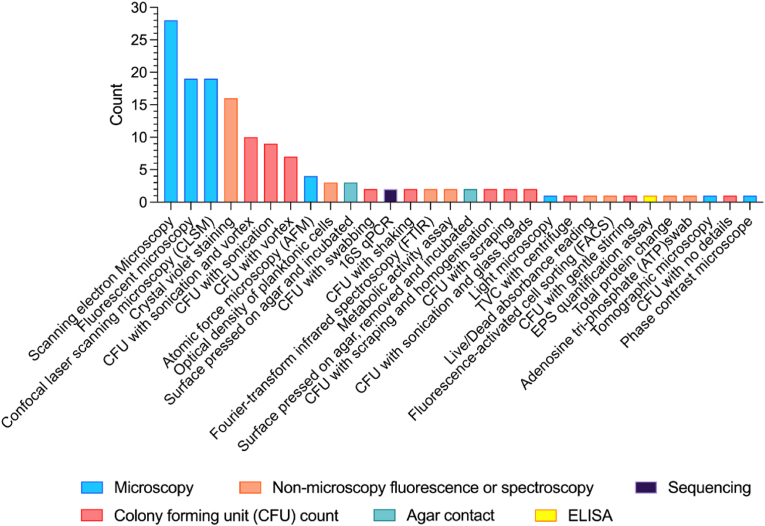
-Methods to assess biofilm formation during the assessment of antibiofilm surfaces. Methods were further categorised into six overarching methodological approaches, which are visualised by the colour of each bar, described in the legend.

Methods to assess the efficacy of antibiofilm action were collated into six approaches within which there were 30 different methodologies employed (Fig. 5). The six approaches consisted of methods that quantified by colony forming unit counts (37%, n = 11), methods using non-microscopy fluorescence or spectroscopy (27%, n = 8), microscopy analysis (23%, n = 7), direct agar-contact (7%, n = 2) sequencing (3%, n = 1), and ELISA (3%, n = 1).

Of all methods across these six approaches, the three most common methods were all microscopy-based, with scanning electron microscopy (SEM, 19%, n = 28) used most often, followed by fluorescent microscopy (13%, n = 19) and confocal laser scanning microscopy (CLSM, 13%, n = 19). Less common microscopy methods included AFM (2.7%, n = 4), light microscopy (0.7%, n = 1), tomographic microscopy (0.7%, n = 1) and phase contrast microscopy (0.7%, n = 1). Where a stain was used (n = 28), the vast majority used Live/Dead (64%, n = 18), followed by DAPI (7%, n = 2), or microorganisms with GFP-labelling or autofluorescence (7%, n = 2). Five other stains (or combinations of stains) were used only once each (4%, n = 1), including Acridine Orange; Propidium Iodide; SYTO 9 and Congo Red; Live/Dead, Texas-Red and SYBR; Live/Dead and SYPRO Ruby. Of all the methods that used microscopy, only ten articles described how they generated data from the microscopic examination. For example, the most common approach (n = 5) was to take 3 images randomly across a sample, with others capturing 20 images (n = 3), 10 images (n = 1) or 5 images (n = 1) across a sample.

Of the non-microscopy methods that used fluorescence or spectroscopy, crystal violet (CV) staining was by far the most common (11%, n = 16). However, approaches to CV staining were varied. Whilst half of those quantifying with CV used a 0.1% CV preparation (50%, n = 8), other preparations including 1% CV (13%, n = 2) and the following were noted only once (6.25%, n = 1); 0.04%, 0.06%, 0.3%, 0.4%, 10%. One article did not describe CV preparation. The amount of time for which the CV was left to stain the biofilm was more evenly distributed and included 15 min (32%, n = 5), 10 min (19%, n = 3), 5 min (19%, n = 3), 20 min (13%, n = 2). One article did not provide this information. Three different chemicals, prepared at various concentrations, were used to solubilise CV following biofilm staining; 95% ethanol (38%, n = 6), 33% acetic acid (19%, n = 3), 30% acetic acid (13%, n = 2), 90% ethanol (13%, n = 2), methanol (1%, n = 1), absolute ethanol (1%, n = 1) and 66% acetic acid (1%, n = 1). The most common wavelength with which the optical density of the solubilised CV was measured, was 595 nm (44%, n = 7), followed by 570 nm (25%, n = 4), 590 nm (19%, n = 3), 600 nm (6%, n = 1) and 690 nm (6%, n = 1).

Other less common non-microscopy methods using fluorescence or spectroscopy included measuring optical density of cells in planktonic suspension above a surface during incubation (2%, n = 3), FTIR (1%, n = 2), metabolic activity assay (1%, n = 2), staining with Live/Dead and reading via spectroscopy (1%, n = 1), fluorescence-activated cell sorting (FACS, 1%, n = 1), total protein change assay (1%, n = 1) and using ATP swabs (1%, n = 1).

Colony forming unit (CFU) counts were also commonly used. Whilst these methods had consistent features, for example, ultimately incubating a dilution of recovered microorganisms onto agar for CFU counts, there were still variations in how the biofilm was recovered/removed from the surface. The most common approach to recovery was to use sonication and vortex mixing combined (7%, n = 10), sonication on its own (6%, n = 9), and vortex mixing on its own (5%, n = 7). Other approaches to removing biofilm were swabbing (1%, n = 2), shaking (1%, n = 2), scraping with homogenisation (1%, n = 2), scraping (1%, n = 2), sonicating with glass beads (1%, n = 2), centrifugation (1%, n = 1) and gentle stirring (1%, n = 1). One article did not provide methodological detail for the removal or recovery of biofilm for CFU calculation. Only one article specifically stated the use of a neutralizer (Soya Casein Digest Lecithin Polysorbate Broth - SCDLP), with many methods describing recovering into PBS, saline and standard microbiological media amongst others (Table 3). Only two articles described assessing the surface after removal of the biofilm to ensure all biofilm had been removed, where in both cases, after sonicating with glass beads, the surfaces were laid on top of agar containing 2,3,5-triphenyltetrazolium chloride dye, where any remaining bacteria were assumed to grow as colonies with a purple colour. Only one article referred to assessing the surface for potential regrowth, however, provides no methodological explanation or data to evidence this.

**Table 3:** Media described as part of recovering for analysis (where appropriate).

Standard microbiology media	*Count*
PBS	15
Saline	5
Not defined	6
Tris buffer	3
500-fold-diluted LB and 4-mm glass beads	2
Liquid media	1
Peptone water	1
BHI broth	1
SCDLP with 1.5 M NaCl	1
Sterile distilled water	1
Sterile tap water	1
Neutralizer/BHI	1
0.9% NaCl-peptone	1

### 3.7 Does an antibiofilm surface have antibiofilm properties?

Of all the articles, only 6% (n = 5) defined requirements for ‘passing’ a surface that possessed antibiofilm efficacy. Of those that did, one article suggested the surface would be considered antibiofilm if it prevented biofilm formation for two weeks. Three articles calculated relative biofilm production on test surfaces compared to control surfaces, with two quantifying using crystal violet optical density readings and one quantifying with CFU counts, where relative biofilm production of less than 100% was considered antibiofilm. One article defined antibiofilm success as achieving the pass criteria of JIS Z 2801 (which is usually determined by the interested parties on an application-by-application basis). Four articles used the term ‘bacteriostatic’ when describing efficacy of their materials. All articles except for one described the use of negative controls, which were predominantly uncoated/unmodified surfaces. Where the coating was in the form of a potentially antibiofilm paint, commercially available paints were used as negative controls. Positive controls were far less common, for example, using a photocatalytic coating as a positive control where the uncharacterised antibiofilm surface involves a photocatalytic coating combined with a biocide.

## 4 Discussion

The analysis described above demonstrates a growing interest in the efficacy testing of surfaces that have antibiofilm potential, in terms of peer-reviewed publication outputs originating primarily from research laboratories. Results described above can be used to answer the original questions posed in the methods section (Table 4). This demonstrates that whilst literature may appear to be using the same method, it can in fact be variable and can lack detail which makes it difficult to reproduce and compare findings.

**Table 4:** Overview of questions posed in the methodology and answers derived from analysis of articles included in this review.

Question	Answer	Recommendation
Is a definition of biofilm present?	Definitions were predominantly absent or vague.	Defined definitions of biofilm and antibiofilm included.
What type of material is being tested?	Variety of different surface types (e.g. metals, polymers)	Clearly define material using language accessible across disciplines.
What type of antimicrobial action is exhibited, if any?	Variety of biocidal, contact active or antiadhesive.	Clearly define antimicrobial action using language accessible across disciplines.
What method was used to form the biofilm (including environment)?	No consensus approach. Methods varied but predominantly used a submerged and static environment. Within methods, media, time, temperature were all varied.	Carefully consider the intended end-use of antibiofilm surface and select method (and method constituents) accordingly, providing rationale.
What species of microorganism was used?	No consensus approach. Microbial species were varied (sometimes without strain identifiers), but predominant use of bacterial species common in the literature.	Carefully consider if microbial species is relevant to the intended application. Provide rationale and strain information for reproducibility.
How was biofilm quantified?	No consensus approach. Variety of different approaches including colony forming unit counts, microscopy, non-microscopy fluorescence/spectroscopy, direct agar contact, sequencing, and ELISA.	Ensure quantification of biofilm provides rigorous data, and is appropriate regarding the material/antibiofilm action and the biofilm formation method.
Were adequate positive/negative controls used?	Whilst negative controls (non-active surfaces) were often (but not always) described, positive controls were not.	Ensure control materials are well described and used appropriately to qualify antibiofilm effect.
How were bacteria remaining on the surface quantified (if at all)?	Assessing surfaces for remaining microorganisms was almost entirely absent from analysis.	Where appropriate, ensure surfaces are assessed for remaining/adhered microorganisms. These might otherwise have been assumed to have been killed, or removed and quantified.
Was regrowth of biofilm assessed?	Assessing surfaces for biofilm growth was almost entirely absent from analysis.	Where appropriate, ensure surfaces are assessed for regrowth of biofilm. Otherwise the surface may have been assumed to be free of residual cells and biofilm.
Does the article claim success for an antibiofilm surface?	No consensus approach. A variety of different vocabulary was used to describe successful antibiofilm activity.	Ensure that if an antibiofilm claim is being made, it is clearly supported by the data presented in the article.
What are the requirements for passing as an anti-biofilm material?	Requirements required of the data to determine antibiofilm were rarely defined.	Careful consideration of requirements which must be met (using robust methodology) to clearly show antibiofilm activity compared to control.
Did the experiment contain repeats or replicates and how were they described?	No consensus approach. The use of, and number of repeats and replicates varied.	Carefully consider the most appropriate number of repeats and replicates to ensure suitable statistical analysis.

**Overall recommendation**: consensus on approach to testing antibiofilm surfaces is required within all disciplines involved in their study.

### 4.1 Variation in methodology

The variety of methods used to produce and quantify biofilm to model different applications/environments has been discussed in the literature before [e.g. 99,100]. However, critical understanding of the wide range of, and variation within, methods that are not standardised (and the potential implication this variation has on reproducibility) has not been implemented. As with all microbiology methods, every methodological choice made can have consequence regarding biological (and data) variation. It is widely agreed in the literature that microorganisms that are commonly associated with human health grow optimally at 35 °C–37 °C, within the range of a healthy internal human body. As such, using these temperatures is logical if the purpose of the experiment is to form an optimum biofilm for these specific strains (and if the intended end-use is directed towards the human body). Such was the choice for most articles featured in this review. However, the literature also shows that incubation temperature can change biofilm phenotype [e.g. 101,102]. Therefore to ensure antibiofilm surface efficacy data are representative of activity in an intended end-use, it is prudent to design experiments using temperatures defined within those end-use environments. Of the articles analysed in this review, only three explicitly rationalised their choice of incubation temperature, which were all focused on food contact surfaces, noting alignment to EU regulation.

Similar consideration should be applied to selection of growth media and microbial strain. The variation in bacterial strain (and even variation of isolates of particular species) selection was significant, featuring isolates already described in existing standards for biofilm (*Staphylococcus epidermidis* ATCC 35984 [103]) and antimicrobial surface testing (e.g. *E. coli* ATCC 8739, *S. aureus* ATCC 6538 P [104]), as well as those that are often used for other biological assays such as *E. coli* DH5α, a well-established engineered strain for plasmid production [105]. Other bacterial strains were likely selected for appropriateness to end-use (e.g. *Listeria monocytogenes*, a well-known foodborne pathogen able to survive in realistic food preparation environmental conditions [106]). Whilst it may be possible that strain selection is dictated by end-use appropriateness, it may also be conceivable that researchers are restricted to what they have available in their lab (e.g. chemistry and engineering facilities may be limited to BSL level 1 microorganisms), which may result in a significant variation in strain usage within the literature. However, given the knowledge that different isolates of the same microorganism can produce biofilm in varying amounts [102] it is not possible to assume that all members of a particular microbial species perform in the same way, nor any potential interaction between species in a multispecies biofilm.

The selection of growth media described in this review reveals similar variation. Importantly, the availability of nutrients has been linked to biofilm formation [107,108], with key differences between high nutrient and low nutrient availability [109,110], which are of course important factors when considering end-use environments. Particularly in the built environment, the optimum nutrients for a microbial biofilm are unlikely to be available. Some media selection is clearly linked to end-use, e.g. synthetic tap water, but most articles described above used nutrient-rich media. Pre-conditioning surfaces with contaminating material (microorganism-free) likely to be found in end-use (known as conditioning films) was widely absent from these articles, but have been shown to be important in biofilm formation [111,112] and will likely interact with efficacy of an antimicrobial surface [113]. Given that there is no clear end-use scenario provided in the publications to support these media selections, it may be that these media are easy to obtain, cheap, easy to prepare. Additionally, it may be that authors who are not microbiologists are more familiar with common media described in the literature and/or may decide to use common media for initial testing.

Across all of these methodological considerations, whilst some were dominated by common choices (e.g. temperature was predominantly 37 °C), the variety of environmental conditions and methodological choices made was significant. Whilst these variations may be beneficial, such as selecting a wider range of end-use appropriate conditions, the variation across the literature makes like-for-like comparisons of antibiofilm surface technologies and efficacy difficult, and creates barriers to reproducible science. Widely agreed and adopted approaches or guidance on how to select and incorporate end-use specific conditions, alongside minimum reporting guidelines may benefit the literature in this respect.

### 4.2 Growing biofilm

Critical to interpreting efficacy of an antibiofilm surface is the formation of a reproducible biofilm. The most common approach to forming biofilm in this analysis was described as a static, submerged system, based on the widely used microtitre plate method. There have been various descriptions of this method in the literature to form bacterial [114,115] and fungal [116] biofilm, and it has been demonstrated to be a repeatable, versatile and easy-to-use method across interlaboratory studies [17]. This approach to biofilm formation is widely used and its design enables users to quantitatively evaluate susceptibility testing of antibiofilm compounds/chemicals in suspension but is not designed to mimic end-use conditions of an antibiofilm surface. Whilst efficacy data for antibiofilm surfaces would ideally be based on methodology that produced a biofilm appropriate for end-use of the antibiofilm surface, for example, if the end-use was a built-environment surface then testing in a non-submerged system would be beneficial, we believe that such widely accepted and used methods such as submerged biofilm formation provide a sensible initial analysis option, with subsequent studies using more appropriate methodology.

Other approaches to growing biofilm were informed by existing standardised methods and well described models. Two widely-used standards for antimicrobial surface efficacy testing, ISO 22196/JIS Z 2801 and ISO 27447 were used in some of the papers that were analysed. ISO 22196 [104] requires the users to inoculate a potential antimicrobial surface with microorganisms in small volumes (100 μL–400 μL), purposefully spreading the inoculum over the surface for maximum surface area and incubating in optimum conditions for the microorganisms (37 °C, >90%) for 24 h. ISO 27447 [117] follows a similar process, but is designed for photocatalytic coatings, and therefore incubates under a UV light. Such methods for antimicrobial efficacy do not provide realistic end-use data [118], and whilst indication of antimicrobial efficacy against microorganisms in liquid culture is useful, it does not necessarily translate into antibiofilm efficacy, particularly for applications in non-submerged environments. This demonstrates two issues relating to standardisation of biofilm research, (i) potentially inappropriate selection of methodology and (ii) lack of standardised biofilm methodology that provides data in support of intended end-use applications. The standards described above are well used in the antimicrobial surfaces research field, and therefore may appear a logical approach for those interested in antibiofilm activity. Critically, as an agreed consensus on the definition of antibiofilm, or agreed methodological approach on generating antibiofilm data are yet to emerge from the wider field, it is perhaps unsurprising that some researchers may rely on well-established standardised methods, assuming appropriateness to their application, particularly if their field of expertise is on the engineering or chemistry development of antimicrobial coatings and surfaces. However, selection of these methods needs careful consideration, as their appropriateness is dependent on the research aim of the particular study. Additionally, this further highlights the need to develop standardised methods for biofilm research, which is currently under active development in various research groups and standard setting organisations globally.

The CDC reactor [119], a well-established model for biofilm formation in a submerged, high shear environment was also described. Originally designed to test biocides against reproducible biofilms formed on coupons, this model may provide antibiofilm efficacy of a surface for applications in submerged flow systems (e.g. pipes) but not in the non-submerged/built environment. Other well-known biofilm models such as the Robbins device [120] (a linear flow system containing multiple coupons upon which biofilm can form) were absent from the analysis. Other flow cell systems reported in this analysis were predominately designed for microscopy analysis *in vitro* [121]. However, use of flow systems, whilst capable of producing reproducible biofilm, need careful consideration, for example incubating biocidal surfaces which may leach active materials and affect non-active controls in the same system. Similarly, the microtitre plate method has been demonstrated to be reliable and reproducible [17], but the articles in this review demonstrate variation in the detail of this method. The well-established drip flow reactor [122,123] which was designed for medical applications, does not keep a sample submerged over time, and has individual cells for each sample ensuring no cross-sample leaching, may provide more appropriate efficacy data of antibiofilm surfaces, but did not feature in the articles analysed for this study. In addition, some of these devices may not be widely available to all laboratories, and not all personnel will be familiar with their nuances. More broadly, a lack of high-throughput methodology is apparent.

### 4.3 Qualitative and quantitative analysis of biofilm

The methods to qualitatively and/or quantitively assess biofilm formation on antibiofilm surfaces were varied, even where the same approach was employed across different articles within this review. For example, even where dilution and plate count procedures are used, the diluent may or may not contain neutralizer: whilst it might be argued that neutralizer is unnecessary because the dilution process itself will effectively remove any active antimicrobial agents, acknowledgement or clarification of neutralizers were almost entirely absent. Articles that use stains and spectrophotometric analysis often used crystal violet, but often at a variety of different concentrations and use different concentrations of different solutions to solubilise for reading, which have been shown to provide variable biofilm quantification [124]. Additionally, where a method is using some element of mechanical action to remove biofilm from a surface for quantification, such as sonication, vortex or both, were often deployed with varying time requirements, all of which may provide variability in biofilm recovery. Microscopy is widely used in the articles described in this review, and indeed across the biofilm literature. There are many different approaches that can be used to quantify biofilm by microscopy including bespoke software options [100,125], but this review demonstrates that often researchers may opt to using images where biofilm is absent in lieu of quantitative data. Whilst this type of image is of value, due to the nature of microscopy only showing a very small portion of a surface, it is important that researchers provide robust methodology to describe how much of a surface they analysed, which is critical to understanding the scale of antimicrobial effect. This is particularly important when examining a surface directly where residual cells are very low in number and only a small area is visible via the microscope. Advice should be sought from a statistician regarding adequate number of test samples, and careful consideration should be given to the types of repeats that are undertaken, and the way they are described, as this vocabulary may be inadvertently used incorrectly further adding to the difficulty of comparing data across different studies. It is essential to check a test surface if biofilm/cells are removed for subsequent indirect quantification, to ensure that the removal method is effective, and numerous replicate microscopic fields should be examined. If the test surface has a propensity to retain cells, for example by topographic features [126], then the facility for subsequent regrowth is enhanced. Even if cells are inactivated by the surface, the ‘dead’ remains might enhance subsequent cell attachment or impair the antimicrobial effect. In this review, none of the papers described a method for examining surfaces post-test. In our opinion, this is a significant omission.

Challenges associated with reproducible science and efficacy assessment of antibiofilm surfaces – definitions, lack of application specific methods, controls, and detail.

It is essential for reproducibility of results within any scientific discipline that the study subject and its intervention are both well-defined. Whilst in many microbiology disciplines this can be relatively straightforward (e.g. studying particular microorganisms), biofilm provides some unique challenges. Generally, biofilm has been considered “aggregates of microorganisms in which cells are embedded in a self-produced matrix of extracellular polymeric substances (EPS) that are adherent to each other and/or a surface” [127,128], with recent discussion acknowledging non-surface associated biofilms [7] and the importance of the interface at which biofilm is forming. However, it is widely accepted that biofilms form differently in different environments (e.g. flow vs static), and as such a biofilm in one environment may be biologically and structurally different from another. This is further complicated by the definition of ‘antibiofilm’, i.e. what is a particular study attempting to demonstrate: curative or preventative action, antiadhesion properties, keeping the number of microorganisms on a surface below a critical threshold (management and control), and so on. The articles analysed in this review demonstrate that authors reporting antibiofilm surfaces in the literature are not clearly or explicitly defining either biofilm or antibiofilm, and in some cases not defining either. Whilst agreed definitions for biofilm and antibiofilm would be beneficial to the literature, at minimum each manuscript should be clearly defining their own study object (biofilm) and action/intervention (antibiofilm) to allow readers to fully assess the validity of methods and associated data and providing direction for regulatory decision making.

Some articles in this review did not report microbial strain, temperature, growth time, sampling strategy, quantification approaches and so on. Interestingly, given that most papers described in this analysis were published in chemistry and engineering-focused journals, and that fundamentally, the advances being described (antibiofilm surfaces) are chemistry and engineering in nature, it may be assumed that those designing the antibiofilm efficacy methodologies may not be microbiologists. If this were the be the case, it is understandable why specific microbiological methodology may be lacking and demonstrates a need for biofilm researchers to engage and educate around these applied areas. Methods such as submerged static biofilm formation are well known and accessible methods described in the wider biofilm literature. As such, non-microbiologists searching the literature may find and modify/adopt these methods to assess efficacy despite their design not being appropriate for the intended application of their antibiofilm surface. Additional methodological expectations such as sufficient use of control surfaces, either negative (no antimicrobial additive or surface modification) or positive (widely accepted or competitor antimicrobial surface) also need careful consideration. Whilst this analysis demonstrates appropriate use of negative controls, positive controls are almost entirely absent, perhaps due to the difficulty in identifying such a material.

Durability of surfaces, the assessment of efficacy over time, with wear (e.g. repeated touch, cleaning regimes), does not form part of the standard methodology described in many antibiofilm surfaces studies (although it is not totally absent from the literature [e.g. 70]), with more recognition of its importance in antimicrobial surfaces literature [129] and US EPA guidance ‘Interim method for evaluating the efficacy of antimicrobial surface coatings’. Similarly, during antibiofilm efficacy testing, understanding biofilm regrowth after quantification/removal could be another indicator of antibiofilm efficacy. Regrowth has been demonstrated on surfaces after treatment with disinfectants or biocides in solution [130], but further research is needed to understand its impact for antibiofilm surfaces.

To ensure that the literature on antibiofilm surfaces are as reproducible as possible, it would be useful to ensure that some guidance on the intricacies of antibiofilm efficacy testing and its impact on method development, alongside widely agreed minimum reporting requirements to support such data and claim, are provided for both researchers as well as journal editorial boards and peer-reviewers.

## 5 Conclusion

Whilst the field of biofilm is rooted in microbiology, and therefore one might expect more literature on antibiofilm surfaces in microbiology-focused journals, the production of an antibiofilm surface is fundamentally an engineering and chemistry challenge, and therefore reported in these discipline-norm journals. However, the biological understanding that is needed to appraise methodological choices when dealing with biofilm (growing or quantifying) should not be understated, as demonstrated by the wide range of methods described above. As the growth rate of publications suggests the number of publications on antibiofilm surfaces is likely to continue growing, it is essential that interdisciplinary teams collaborate to ensure that the methods used to generate both the engineering/chemistry and biological data that support claims of antibiofilm activity are appropriate.

This analysis demonstrates that researchers may benefit from considering the relationship with their experimental design and intended antibiofilm surface applications, likely reducing the use of rich media and using more realistic temperatures (unless using as a comparison with existing literature). Additionally careful consideration of microbial strains and appropriate methodological choices in growing and assessing biofilm is important, and at minimum, thorough reporting of methodological decisions is essential. Without an interdisciplinary approach, those conducting antibiofilm efficacy testing of surfaces run the risk of using methods that may be less appropriate, which as the literature grows may become embedded and used more often. It is also clear that the field would benefit from developing standardised methods as well as minimum reporting guidelines and an established definition of antibiofilm activity, coupled with increased cross-disciplinary collaboration to ensure both the methodological choices and associated reproducibility are both described appropriately in the literature.

## CRediT authorship contribution statement

**J. Redfern:** Writing – original draft, Visualization, Methodology, Investigation, Funding acquisition, Formal analysis, Data curation, Conceptualization. **A.J. Cunliffe:** Writing – review & editing, Investigation, Formal analysis, Data curation. **D.M. Goeres:** Writing – review & editing, Methodology, Investigation, Formal analysis, Data curation, Conceptualization. **N.F. Azevedo:** Writing – review & editing, Methodology, Formal analysis, Data curation, Conceptualization. **J. Verran:** Writing – review & editing, Formal analysis, Data curation, Conceptualization.

## Declaration of competing interest

The authors declare the following financial interests/personal relationships which may be considered as potential competing interests:

Darla Goeres is senior editor of the journal ‘Biofilm’. Nuno Azevedo is a member of the editorial board for the journal ‘Biofilm’. If there are other authors, they declare that they have no known competing financial interests or personal relationships that could have appeared to influence the work reported in this paper.

## Data Availability

All data is presented within the article.
